# What "Panel-Prying" Really Is

**Published:** 1921-01-15

**Authors:** 


THE PUBLIC WELL-BEING—(*continued*).

### What "Panel-Prying" Really Is.

While in these columns our views have already been expressed as to the necessity for ensuring that the confidence between doctor and patient shall be carefully preserved in the new medical record system just established for panel patients, we cannot help thinking that the outcry in the daily press has been made on a note of extreme exaggeration. *The Times* headed the pack with a savage growl of anger and alarm, and now all the little dogs of the press are snapping for all they are worth at the Ministry of Health, and one might almost assume, from the state of hysteria which is indicated, that in future all the ailments of all panel patients will be publicly placarded up and down the country for the delectation of a scandal-mongering and curious world.

In fairness to the Ministry, let us admit that they have devised what, at any rate, appear to be ample safeguards, though we still think it would be a judicious procedure to invite the considered criticisms and suggestions of experienced panel doctors in the large towns. This might at least tend to allay whatever public excitement has been fomented by subjecting the scheme to practical analysis. In an interview the other day a Ministry of Health official defended the plan on many points of detailed criticism directed against it by correspondents in the press, particularly by members of the public who have not made themselves familiar with the actual scheme.

With regard, for example, to the contention that insurance committee clerks will be able (and anxious) to learn the nature of the illnesses of patients and to gloat over it and pass the information on to others, the official in question declared that an evil-minded clerk will have no such opportunity. The new card, he points out, has on the front the patient's name, address, membership number, etc. On the back are the panel doctor's remarks. If a man is changing from one district to another the card is put by the doctor into an envelope, with its front facing the front of the envelope, and gummed up and sent to the local insurance committee. On the front of the envelope is an aperture through which can be read the name, etc. The back of the card is hidden from the eyes of the clerks at the insurance committee's office, and cannot be read without tearing open the envelope. The card is despatched in its envelope to the insurance committee in the man's new district, stamped, and handed intact to the panel doctor.

There is, however, the Regional Medical Officer, employing one or two clerks, who acts as consultant, adviser, and referee for the panel doctors in his district. It is possible—and we should say it would be a common occurrence—that he may call upon them to send in their cards, in order, for example, to ascertain the amount of influenza in his district. In that case his clerks will undoubtedly read the doctor's comments. But these clerks, unlike the insurance committee's clerks, are Civil Servants, who are bound by the Official Secrets Act. If they divulge anything they are liable to seven years' penal servitude, or some such severe sentence. The panel patient's medical history is, therefore, in the opinion of the Health Ministry's advisers, in safe keeping. It is, of course, possible, even with these admittedly careful safeguards, that leakage may occur here and there and now and again from one of these connecting channels, but, on the whole, we think it is improbable, and that in any case it would be an unusual occurrence. The system is now in operation, and it may be taken for granted that if experience shows that even a moderate amount of leakage takes place public opinion will compel the Ministry to withdraw a plan that is undoubtedly designed to assist research and to give value to medical statistics.

